# Supplementary figures and images for: Exploring regional air pollution transition dynamics: A multi-state markov model approach
Source: PLoS One. 2025 Oct 7;20(10):e0333849. doi: 10.1371/journal.pone.0333849 (PMC12503330; doi:10.1371/journal.pone.0333849)

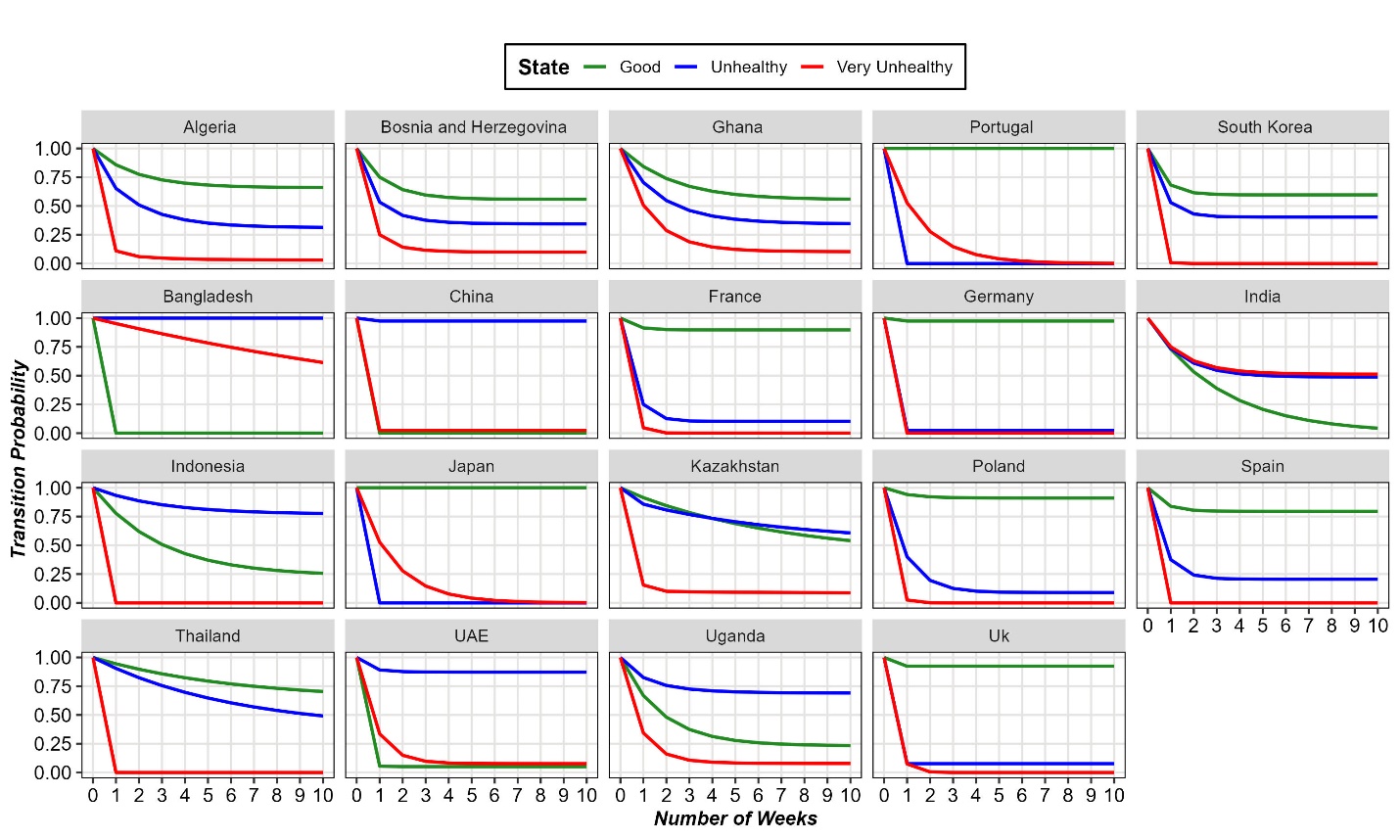


**Supporting Figure 1: Transition probability of weekly AQI categories across 19 countries.**

Supplement: S1 Fig — (DOCX) [file pone.0333849.s001.docx]
