# Supplementary material for: Exploring regional air pollution transition dynamics: A multi-state markov model approach
Source: PLoS One. 2025 Oct 7;20(10):e0333849. doi: 10.1371/journal.pone.0333849 (PMC12503330; doi:10.1371/journal.pone.0333849)
Supplement: Attachment — Submitted filename: PONE -D-25-21399.pdf [file pone.0333849.s002.pdf]

PONE-D-25-21399

Exploring Regional Air Pollution Transition Dynamics:

A Multi-State Markov Model Approach

Study highlights are the following :

- ❖ Data and statistics used in the study are recent
- ❖ It is a very interesting thematic topic related to the SDG, which is usually explored and discussed by authors given the importance of subjects such as pollution and global health.
- ❖ A well selected econometric tool.

Shortcomings

- ❖ *Form problems*

-In general , figures and tables are accompanied by source .

-The introduction and the literature review are mixed . The general structure of an article or research paper should contain section 1 . introduction, section 2. Literature review , section 3. Methodology and sampling , section 4. Findings…..

-The study plan is not mentioned in any part of the document ; generally it must be appear in the introduction .

-Titles and subtitles are not well formulated ; for example L 116 should be replaced by sampling; this term summarizes all information's related to the size of sample, variables. Consequently subtitles in L 117 and L 129 will be deleted . The same remark about L154 : to change the title by "methodology". Or the paragraph can be inserted as a suite for the section 3 methodology and sampling ; in this case section 3 corresponds to L116 – L187.

-About graphics, in the figure N2 , there are 3 diagrams : they should be presented separately with their titles and not pooled . Either these graphics should be interpreted by taking in consideration both dimensions temporal and individuals (country) .

Empirical problems

-Regarding the 19 countries used in the sampling were not cited or enumerated in the article ; only regions were distinguished . countries name were only mentioned in the graphs of each region . So , the author can add them in an appendix for example

-The computing formula of Thahelm (2019) was not developed or defined just referred in the text (L122- 123). The applied methodology in this research paper is not well expressed.

- The econometrical methodology used is the multistate model Markov, so the author have to check the "Time Homogenous Model" (THM) .

-The presentation of table n1 is poor. The columns contain data and rates; only one value is given. Here, it is a qualitative variable. If the table is transformed into a circular diagram, it will be more suitable. In this way, the information will be simpler and clearer, making it easier to understand, and also making it easier to compare between regions.

Discussions :

- The author must focus more on the prediction and suggestions sections, which are not developed enough. They need to provide more information on the various possible scenarios and suggestions by region.

-Main findings are briefly given in the conclusion
