# Supplementary material for: Exploring regional air pollution transition dynamics: A multi-state markov model approach
Source: PLoS One. 2025 Oct 7;20(10):e0333849. doi: 10.1371/journal.pone.0333849 (PMC12503330; doi:10.1371/journal.pone.0333849)
Supplement: Attachment — Submitted filename: PONE -D-25-21399.R1.pdf [file pone.0333849.s004.pdf]

PONE-D-25-21399R1

Exploring Regional Air Pollution Transition Dynamics:

A Multi-State Markov Model Approach

The authors have addressed the majority of the reviewer's comments and made the necessary revisions. However, two points remain insufficiently resolved:

1. **Sources under tables and figures**: While the issue of sources has been partially clarified, the convention requires that the source be specified directly below each table or figure, aligned to the right. Please indicate the source as follows:
    - *Source: Author's calculation* (if it is the result of the author's computations);
    - *Source: Author's compilation* (if the table or figure has been compiled by the author from different references);
    - *Source: [Reference]* (if it is directly taken from another work).
2. **Figures – Titles and Presentation**
   About graphics, in the figure N2 , there are 3 diagrams : they should be presented separately with their titles and not pooled . Either these graphics should be interpreted by taking in consideration both dimensions temporal and individuals (country) .

In Figure N°2, three separate diagrams are presented together under a single title. For clarity and precision, they should be **presented separately**, with each diagram accompanied by its own specific title ,example ***"Weekly Average AQI for African Countries")***

Each diagram, should be interpreted separately , explicitly considering both dimensions: temporal evolution and country-specific differences .
